# Second magnetization peak, rhombic-to-square Bragg vortex glass transition, and intersecting magnetic hysteresis curves in overdoped BaFe_2_(As_1−*x*_P_*x*_)_2_ single crystals

**DOI:** 10.1038/s41598-020-74156-z

**Published:** 2020-10-14

**Authors:** L. Miu, A. M. Ionescu, D. Miu, M. Burdusel, P. Badica, D. Batalu, A. Crisan

**Affiliations:** 1 National Institute of Materials Physics, 077125 Magurele, Romania; 2 National Institute of Laser, Plasma, and Radiation Physics, 077125 Magurele, Romania; 3 SC Fileo Buildup SRL, 060816 Bucharest, Romania

**Keywords:** Materials science, Physics

## Abstract

The second magnetization peak (SMP) in the fourfold symmetric superconducting single crystals (such as iron pnictides and tetragonal cuprates) has been attributed to the rhombic-to-square transition (RST) of the quasi-ordered vortex solid (the Bragg vortex glass, BVG). This represents an alternative to the pinning-induced BVG disordering as the actual SMP mechanism. The analysis of the magnetic response of BaFe_2_(As_1−*x*_P_*x*_)_2_ specimens presented here shows that the SMP is not generated by the RST. However, the latter can affect the pinning-dependent SMP onset field if this is close to the (intrinsic) RST line, through the occurrence of a “shoulder” on the magnetic hysteresis curves *m*(*H*), and a maximum in the temperature variation of the DC critical current density. These features disappear in AC conditions, where the vortex system is dynamically ordered in the RST domain, emphasizing the essential role of vortex dislocations for an efficient accommodation of the vortex system to the pinning landscape and the SMP development. The *m*(*H*) shoulder is associated with a precipitous pinning-induced proliferation of dislocations at the RST, where the BVG elastic “squash” modulus softens. The DC magnetization relaxation indicates that the pinning-induced vortex system disordering continues above the RST domain, as the basic SMP mechanism.

## Introduction

Vortex pinning enhancement at high external magnetic fields *H* in superconducting single crystals with randomly distributed vortex pinning centres is one of the most relevant aspects for the understanding of the vortex-phase diagram of superconductors. In the case of weakly pinned specimens, one has the so called peak effect^1–6^, where the maximum in the magnetic field dependence of the critical current density *J*_c_ (proportional to the irreversible magnetic moment) is rather sharp and located close to the DC irreversibility line (defined in the magnetic field-temperature *T* plane by a vanishing irreversible magnetization).

When vortex pinning is stronger, the peak effect is substituted by a wide second magnetization peak (SMP) in increasing *H*, with the onset field *H*_on_ and the peak field *H*_p_ far below the irreversibility line, leading to fishtail-shaped DC magnetic hysteresis *m*(*H*) curves. Among many SMP mechanisms and models (see, for example,^7–20^), a pinning-induced disordering of the low-*H* quasi-ordered vortex solid (the Bragg vortex glass^21,22^, BVG, stable against dislocation formation) has been considered to be at the origin of the SMP for various superconducting single crystals^9,14–18^.

Significant progress for the understanding of the vortex phase diagram and the occurrence of the SMP has been made by considering the competition between the thermal energy, the pinning energy, and the elastic energy in the vortex system^11^. In the low-*T* domain, the thermal energy can be neglected, and the simple energy balance relation leads to an onset field which is independent of temperature. This is also the result of the order–disorder transition derived in Ref.^12^. It has been later shown^14^ that the upward curvature of the *H*_on_(*T*) variation in the low-*T* range can be explained by taking into account the reduction of the effective pinning energy at low temperatures, where the probing current density is closer to *J*_c_. This approach was suggested by the often observed time evolution of the SMP, with the characteristic fields decreasing at high relaxation levels. Since the SMP extends over a large magnetic field interval, it has been proposed that the pinning-induced BVG disordering is continuous^15,17^, starting at *H*_on_, where the energy for the plastic vortex deformation^23^ is smaller than the effective pinning energy, and finishing at *H*_p_, where the vortex system is amorphous. The essential point is the proliferation of dislocations in the vortex system for *H* between *H*_on_ and *H*_p_ (at large scales first), where the pinning increase is caused by the efficient accommodation of vortices to the pinning centres in the presence of vortex dislocations. This idea is strongly supported by the repeatedly reported elastic vortex creep-plastic creep crossover across the SMP^9,24–26^.

Alternatively, is has been argued that in (tetragonal) La_2−*x*_Sr_*x*_CuO_4_ single crystals, with fourfold symmetric inter-vortex interactions, the SMP is the direct result of the characteristic, structural rhombic-to-square transition (RST) of the BVG^13^, in which case the upward curvature in *H*_on_(*T*) at low *T* results directly (see below). It is known for a long time that in certain anisotropic low-*T*_c_ superconductors (such as borocarbides, Nb, and V_3_Si) the vortex solid undergoes the RST, as noted in^13^. Using small-angle neutron scattering experiments, the crossover toward a square vortex arrangement with increasing magnetic field was clearly seen in La_2−*x*_Sr_*x*_CuO_4_^27^, which may reflect the importance of the anisotropic vortex cores in the *d*-wave superconductors. The main aspect is the softening of the BVG elastic “squash” modulus *C*_sq_ at the RST line in the (*H*, *T*) plane^28^, and it has been predicted in Ref.^29^ that this BVG softening should lead to a maximum of the critical current density *J*_c_(*H*, *T*) in the elastic (collective) pinning regime, with

1 $$ J_{{\text{c}}} \propto {1}/C_{{{\text{sq}}}} . $$

However, the vortex pinning in the BVG (rhombic or square) is generally weak, and a better compliance of vortices to the pinning structure in the elastic regime may not be able to generate the observed, pronounced SMP.

The 122-type iron pnictide single crystals in *H* parallel to the crystallographic *c* axis exhibit a well-developed SMP^30,31^, and the above RST-related SMP model has been extended to these fourfold symmetric superconductors, as in the case of Ba(Fe_0.925_Co_0.075_)_2_As_2_^32^ and BaFe_2_(As_0.68_P_0.32_)_2_^33^ (for which the existence of the BVG has been proven^34^). The SMP in LiFeAs (from the 111 family) received the same interpretation^35^. The anisotropic interaction with fourfold symmetry induces a rhombic rather than hexagonal vortex arrangement, which transforms into a square one when the vortex separation decreases. Thermal fluctuations assist in breaking the rhomb symmetry, lowering the transition field *H*_RST_ as the temperature increases. By minimizing the free energy of a square vortex lattice with respect to the elastic moduli, it was found that

2 $$ H_{{{\text{RST}}}} \left( T \right) \propto (T_{0} - T)T^{ - \nu } \lambda^{{{2}({1} - \nu )}} , $$

where *λ* is the London magnetic penetration depth, which is ~ 108 nm in the low-*T* limit for BaFe_2_(As_0.68_P_0.32_)_2_^33^, *T*_0_ is a constant lower than the critical temperature *T*_c_, and the exponent *ν* is close to unity^13^. The location of the RST line relative to *H*_on_ and *H*_p_ is controversial. An exclusive relationship between the SMP and the RST implies the identification of *H*_RST_(*T*) with *H*_p_(*T*), as proposed for La_2−*x*_Sr_*x*_CuO_4_^13^, whereas in the case of Ba(Fe_0.925_Co_0.075_)_2_As_2_ it has been argued^32^ that the RST line should lie between *H*_on_(*T*) and *H*_p_(*T*), where the normalized magnetization relaxation rate has a minimum.

In this context, we recall the interpretation of the SMP in the fourfold symmetric superconductors by analysing the magnetic response of BaFe_2_(As_1−*x*_P_*x*_)_2_ (P-Ba122) single crystals. It was found that the SMP cannot be generated by the (elastic) rhombic-to-square BVG transition, but the RST can influence the SMP onset field if this is close to the intrinsic RST transition field, as revealed for overdoped specimens. In such a situation, the RST manifests itself through the occurrence of a “shoulder” on the magnetic hysteresis curves *m*(*H*), i.e., a relatively rapid increase of the effective pinning with increasing *H* for a limited magnetic field domain located just above *H*_on_. The evolution of the *m*(*H*) shoulder with temperature leads to the intersection of the isothermal magnetic hysteresis curves, and, consequently, to a peak in the temperature variation of the DC critical current density *J*_c_(*T*). The AC magnetic measurements performed by us indicate that when the vortex system is dynamically ordered in the RST domain the above features disappear. This suggests that the *m*(*H*) shoulder is associated with a precipitous proliferation of dislocations in the vortex system on crossing the RST line, where the softening of the BVG elastic squash modulus appears. Analysis of the DC magnetization relaxation shows that the pinning-induced vortex-system disordering continues above the RST range, as the basic mechanism for the occurrence of the SMP in the case of superconductors with fourfold inter-vortex interactions, as well.

## Results and discussion

It is well established that by increasing the P doping level beyond the optimal one (where *T*_c_ is maximum) the critical current density of P-Ba122 single crystals decreases. A quantitative analysis of the *J*_c_(*T*) dependence^31^ indicated that the characteristic pinning mechanisms [related to the spatial variation of *T*_c_ (*δT*_c_ pinning) and the fluctuations in the charge carrier mean free path] are enhanced for optimally doped and underdoped samples. The P-Ba122 specimen thoroughly investigated here is an overdoped single crystal, with the nominal *x* ~ 0.33, denoted below P-Ba122od, which has the critical temperature *T*_c_ = 27.5 K (determined at the onset of the diamagnetic signal, see Methods). This is similar to the single crystal investigated in Ref.^33^, for which the occurrence of the RST has been signalled using DC magnetic measurements. To highlight the specific shape of the magnetic hysteresis curves of overdoped samples, an optimally doped single crystal (P-Ba122op, *x* ~ 0.30, *T*_c_ = 29 K), with stronger pinning, has been considered.

**Figure 1:**
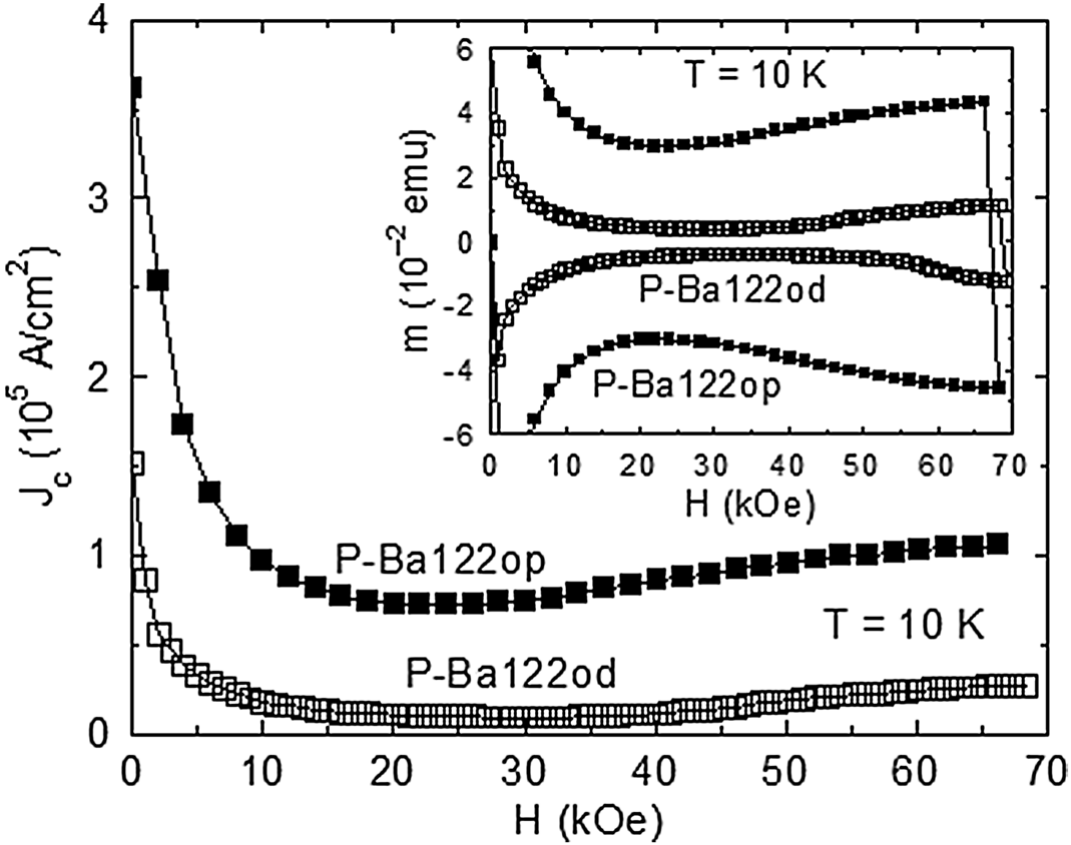
Main panel: Magnetic field *H* variation of the DC critical current density *J*_c_ determined for the optimally doped specimen P-Ba122op and the overdoped single crystal P-Ba122od (of similar dimensions) from the descending branches of the magnetic hysteresis curves *m*(*H*) at the temperature *T* = 10 K plotted in the inset. The particular difference between the DC magnetic hysteresis curves *m*(*H*) from the inset is that in the case of P-Ba122od there is a wide *H* range of weak collective (elastic) pinning in the low-field quasi-ordered vortex solid (the Bragg vortex glass), followed by a precipitous increase of ∣*m*∣ at *H* ~ 60 kOe on the ascending *m*(*H*) branch.

Figure 1 (main panel) illustrates the *J*_c_(*H*) variation for P-Ba122od and P-Ba122op at *T* = 10 K, where *J*_c_ has been determined with the Bean model for rectangular specimens^36^ from the descending branches of the *m*(*H*) curves plotted in the inset. By increasing doping from ~ 0.30 to ~ 0.33, *J*_c_ for *H* around the SMP onset at *T* = 10 K decreases from ~ 7 × 10^4^ to ~ 10^4^ A/cm^2^. The particular difference between the *m*(*H*) curves of P-Ba122od and of the optimally doped specimen P-Ba122op is that in the former case there is a wide magnetic field range of weak elastic (collective) pinning (in the BVG), followed by an increase of ∣*m*∣ around *H* = 60 kOe on the ascending *m*(*H*) branch (see the inset of Fig. 1). We associate below the RST with this precipitous enhancement of the effective pinning in increasing *H* just above *H*_on_.

**Figure 2:**
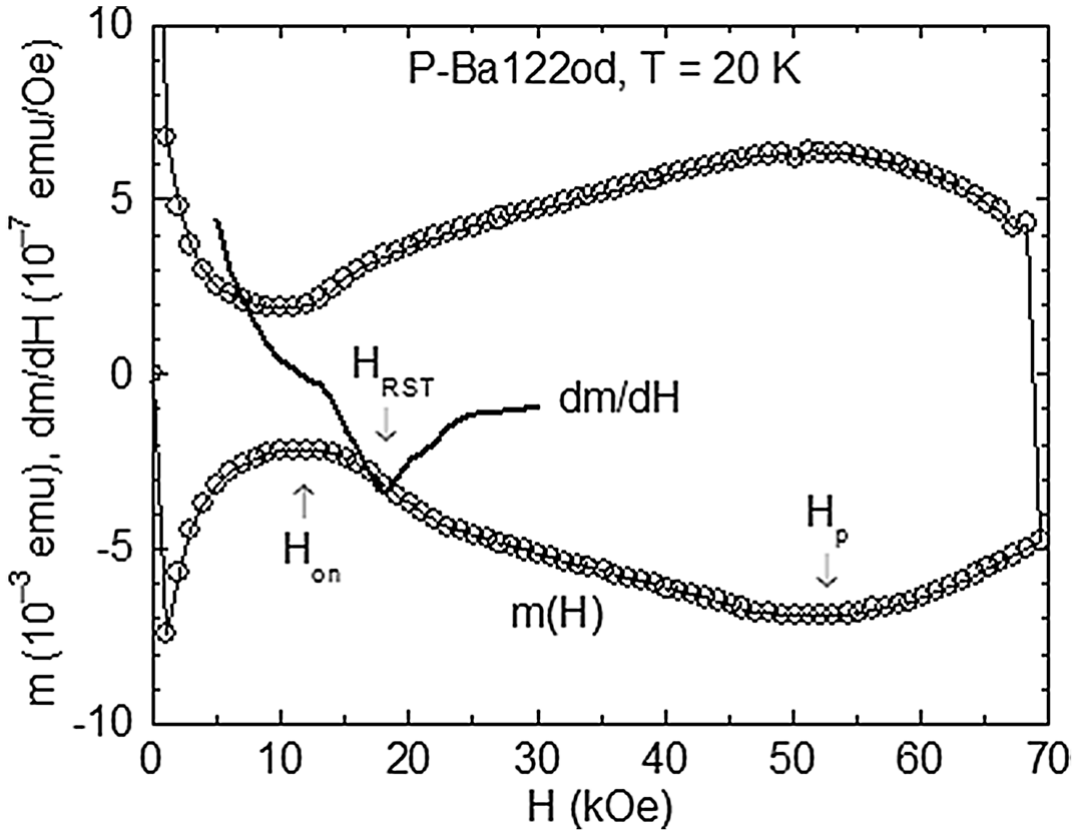
The DC magnetic hysteresis curve *m*(*H*) of P-Ba122od at *T* = 20 K, with the onset field *H*_on_ and the peak field *H*_p_ of the SMP indicated by arrows. We associate the structural rhomb-to-square vortex phase transition with the “step-like” enhancement of ∣*m*∣ in increasing *H*, giving rise to an *m*(*H*) shoulder. The structural transition field *H*_ST_ has been taken at the local minimum of d*m*/d*H* (represented by a continuous line).

The DC magnetic hysteresis curve of P-Ba122od at *T* = 20 K is plotted in Fig. 2, where *H*_on_ and *H*_p_ of the SMP are indicated by arrows. One notes the development of an intriguing *m*(*H*) “shoulder” (leading to “pear-like” shaped hysteresis curves), which was observed for other overdoped P-Ba122 and K-Ba122 specimens^31^. The *m*(*H*) shoulder exhibits history effects. On the descending *m*(*H*) branch, the vortex system seems to remain trapped in more strongly pinned high-*H* states (see the inset of Fig. 1). For this reason, we considered the shoulder on the ascending *m*(*H*) branch, and the transition field *H*_RST_ has been taken at the local minimum of d*m*/d*H*, as illustrated in Fig. 2.

**Figure 3:**
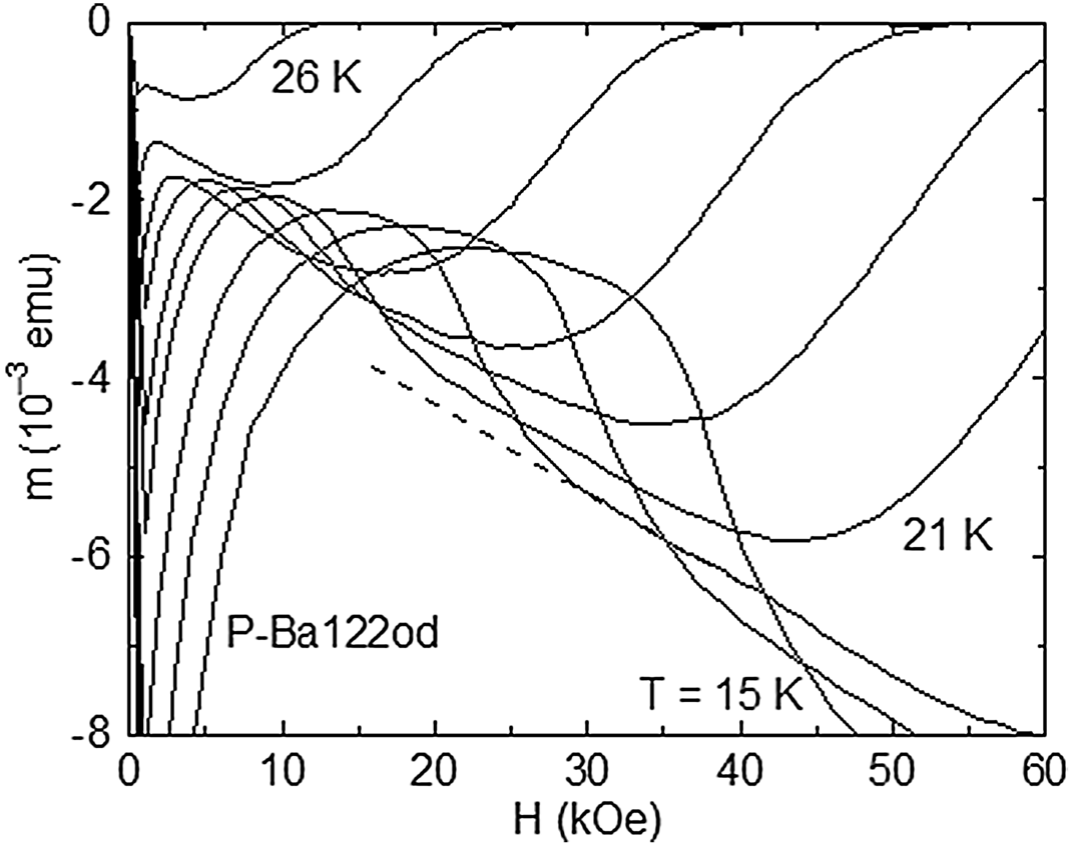
Evolution of the *m*(*H*) shoulder in increasing *H* observed for P-Ba122od with decreasing temperature *T* (from 26 to 21 K with a step of 1 K, and from 21 to 15 K, step of 2 K), leading to the intersection of the *m*(*H*) curves. The dashed line related to the *m*(*H*) curve at *T* = 19 K represents a liner fit of the *m*(*H*) data just above the shoulder, to estimate the width of the associated structural vortex phase transition.

**Figure 4:**
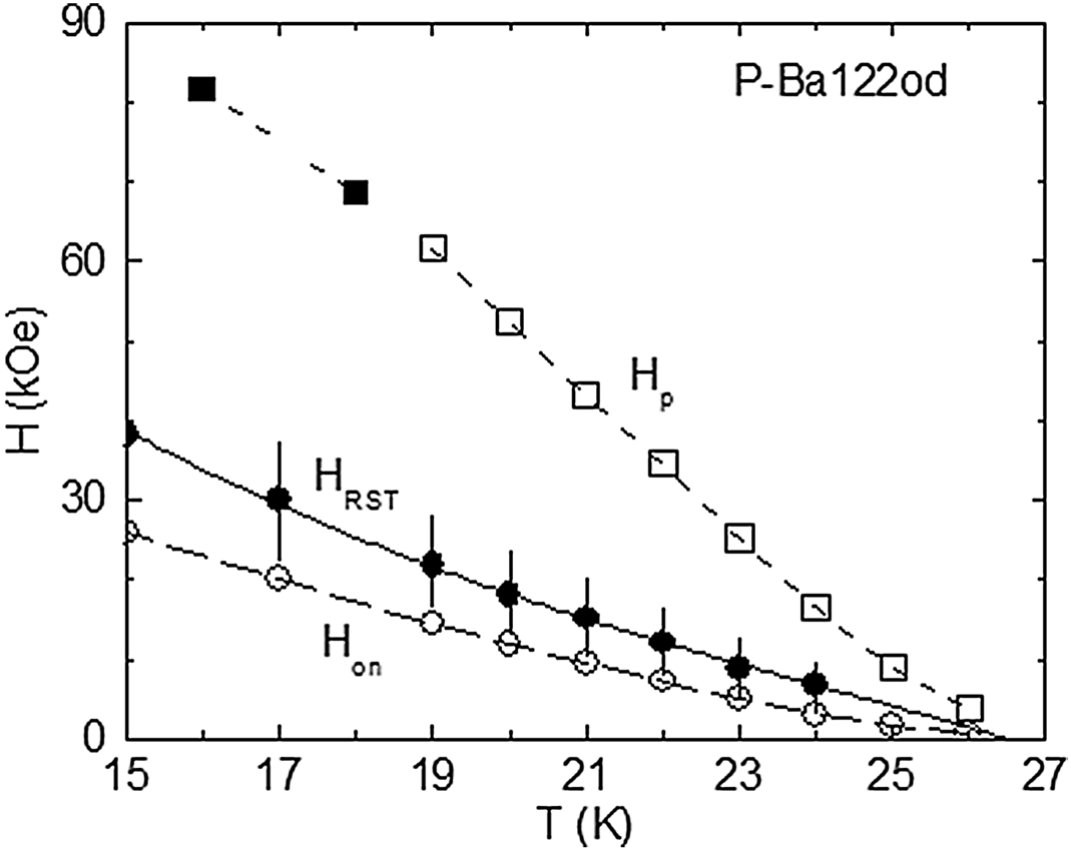
Temperature variation of the structural transition field *H*_ST_ (determined as in Fig. 2), the onset field *H*_on_(*T*), and the peak field *H*_p_(*T*). The vertical segments on *H*_ST_(*T*) illustrate the transition width extracted for several temperatures (by taking the structural transition symmetric relative to *H*_ST_). The continuous line represents the fit of the *H*_ST_(*T*) dependence with (2), by considering the two-fluid model for the temperature variation of the magnetic penetration depth. The filed-symbol *H*_p_(*T*) values have been determined from the magnetic hysteresis curves registered with the PPMS.

The evolution of the *m*(*H*) shoulder with temperature is depicted in Fig. 3. As *T* decreases, the collective pinning in the rhombic BVG of P-Ba122od remains weak and the *m*(*H*) shoulder becomes more pronounced, generating the intersection of the *m*(*H*) curves (Fig. 3). The dashed line on the *m*(*H*) curve at *T* = 19 K illustrates a linear fit of the *m*(*H*) data just above the shoulder, to estimate the transition width. The temperature variation of the transition field *H*_RST_ determined as in Fig. 2, *H*_on_(*T*), and *H*_p_(*T*) are plotted in Fig. 4. The vertical segments on *H*_RST_(*T*) represent the transition width extracted for several *T* values (by considering the RST symmetric relative to *H*_RST_), showing that *H*_on_ is close to the lower *H* edge of the RST. One notes the upward curvature on *H*_RST_(*T*), which is no longer present on *H*_p_(*T*) below ~ 21 K. The *H*_RST_(*T*) dependence determined by us is in agreement with (2), by considering the two-fluid model for *λ*(*T*)^23,33^. The fit of the *H*_RST_(*T*) variation from Fig. 4 supplies *T*_0_ = 26.5 K < *T*_c_, and ν = 0.8 (close to unity), as predicted by the RST theory^13^). At this point, it is worthy to note that if one takes *H*_RST_ as the field value where the shoulder in ∣*m*(*H*)∣ is completed in increasing *H*, it results *T*_0_ > *T*_c_, which is in conflict with (2).

**Figure 5:**
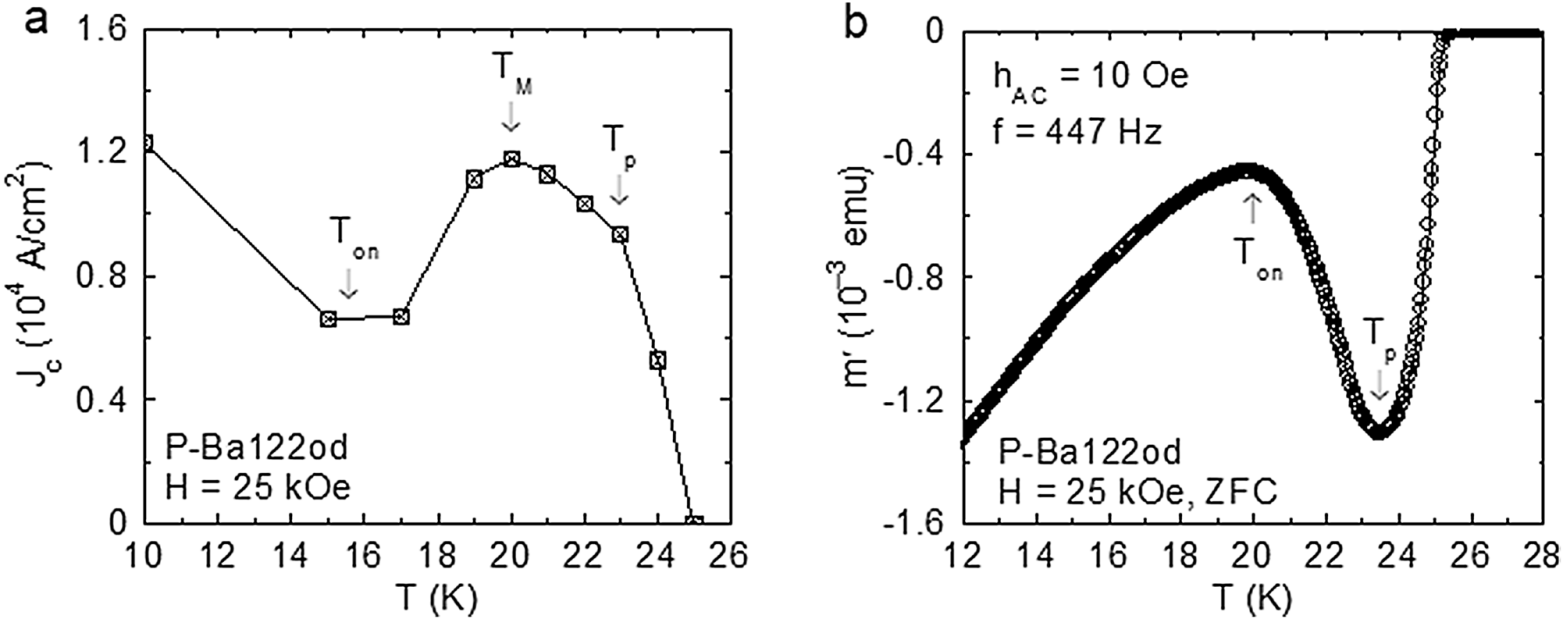
(**a**) Temperature variation of the DC critical current density *J*_c_(*T*) determined with the Bean model using the magnetic moment *m*(*T*) of P-Ba122od in *H* = 25 kOe, with *m* taken from the isothermal magnetic hysteresis curves *m*(*H*) registered at different *T* values (Fig. 3). The DC onset temperature *T*_on_ (around 16 K) and the DC peak temperature *T*_p_ = 23 K represent, in agreement with the *m*(*H*) curves from Fig. 3, the *T* values where the applied field *H* = 25 kOe equals *H*_on_(*T*) and *H*_p_(*T*), respectively. The *J*_c_(*T*) maximum at *T*_M_ = 20 K (between *T*_on_ and *T*_p_) is directly related to the ∣*m*(*T*)∣ values where the *m*(*H*) shoulder is completed, and, due to the large transition width, *T*_M_ is significantly higher that the structural transition temperature *T*_RST_(*H* = 25 kOe) ~ 18 K in Fig. 4. (**b**) Temperature dependence of the in-phase component *m*′ of the AC magnetic moment (directly related to the density of the macroscopic screening currents) for P-Ba122od in *H* = 25 kOe (ZFC, *h*_AC_ = 10 Oe, *f* = 447 Hz). The onset temperature *T*_on_ and the peak temperature *T*_p_ (indicated by arrows) correspond to the SMP generated in the sample region penetrated by the AC critical state (see text). Note that in **b** there is no feature to correspond to the *J*_c_(*T*) maximum at *T*_M_ in **a**.

For intersecting isothermal *m*(*H*) curves, the *m*(*T*) variation obtained for a constant *H* with *m* taken from the magnetic hysteresis curves registered at different *T* values is obviously nonmonotonic, leading to a *J*_c_(*T*) peak, as illustrated in Fig. 5a for *H* = 25 kOe. When *m*(*T*) is directly measured in increasing *T* at a constant *H* applied in ZFC conditions, the nonmonotonic dependence does not appear^33^. This is because the external magnetic field is kept constant, and no inductive processes to bring the screening current density close to the critical value *J*_c_ are present. Thus, by entering a domain of stronger pinning, one can get at most a slowdown of the magnetic relaxation, i.e., a slower decrease of ∣*m*∣ with increasing temperature.

On the *J*_c_(*T*) variation from Fig. 5a one can distinguish the DC SMP onset temperature *T*_on_ around 16 K, and the DC peak temperature *T*_p_ = 23 K, representing, in agreement with the *m*(*H*) curves from Fig. 3, the *T* values where the applied field *H* = 25 kOe equals *H*_on_(*T*) and *H*_p_(*T*), respectively. The *J*_c_(*T*) maximum at *T*_M_ = 20 K (located between *T*_on_ and *T*_p_) is directly related to the ∣*m*(*T*)∣ values where the *m*(*H*) shoulder is completed, and, due to the large transition width, *T*_M_ is significantly higher that *T*_RST_(*H* = 25 kOe) ~ 18 K in Fig. 4.

The main issue is to find out if the *m*(*H*) shoulder [leading to the DC *J*_c_(*T*) maximum at *T*_M_ in Fig. 5a] corresponds, through (1), to the RST in the elastic pinning regime^29^. Alternatively, in the SMP model based on the pinning-induced BVG disordering^14,15,17,18^, this can be the result of a precipitous proliferation of dislocations at the RST, where the BVG elastic squash modulus softens^28^. At this point, the use of AC magnetic measurements becomes helpful, offering the possibility to have an unambiguously ordered (dislocation free) vortex system across the RST domain. The dynamic ordering of the vortex system (i.e., healing of dislocations) at high drives^37,38^ and “shaking” the vortices^39^ has been proven. For this purpose, the presence of the nonlinear AC magnetic response with a large AC critical-state penetration is essential. It has been recently shown^40^ that when pinning is strong and the demagnetization effects are negligible the AC magnetic signal at usual frequencies and amplitudes remains in the linear (Campbell) regime^41^ up to close to the irreversibility line, and the SMP (a bulk phenomenon) does not develop, regardless of the *H* and *T* setting protocol. The situation changes drastically for P-Ba122od, due to the relatively weak pinning and pronounced demagnetization effects in perpendicular magnetic fields, where the AC field amplitude at the sample edge is strongly enhanced. In these conditions, the nonlinear regime with a large AC critical-state penetration is reached far below the irreversibility line. For AC magnetic measurements where *H* is constant, the effective inductive processes during the AC cycles (enhanced by demagnetization effects) keep the screening current density close to the critical value. The SMP is generated in the sample region penetrated by the AC critical state, and the temperature variation of the induced current density is modulated accordingly, as presented below.

Figure 5b illustrates the temperature dependence of the in-phase component *m*′ of the AC magnetic moment (directly related to the density *J* of the macroscopic screening currents^42^) for P-Ba122od in *H* = 25 kOe, obtained with the nominal AC field amplitude *h*_AC_ = 10 Oe and the frequency *f* = 447 Hz. The represented *m*′(*T*) has been registered in the ZFC protocol. Actually, the FCC *m*′(*T*) (not shown) and the ZFC *m*′(*T*) almost overlap, indicating an extended AC critical-state penetration. The onset temperature *T*_on_ ~ 20 K (where the induced *J* and |*m*′| are minimum), and the peak temperature *T*_p_ ~ 23.5 K (where *J* and |*m*′| are maximum) in the used AC conditions correspond to the SMP generated in the sample region penetrated by the AC critical state. By difference with the DC SMP (Fig. 3), the characteristic fields for the AC SMP are shifted to higher values. In the SMP model based on the pinning-induced proliferation of dislocations, this is the manifestation of the dynamic ordering of the vortex system. The ordering effect is large at the onset of the SMP, where the dislocation density is reduced. At *H* = 25 kOe, one has the AC *T*_on_ = 20 K (Fig. 5b), which means that the AC *H*_on_(*T* = 20 K) = 25 kOe. This is considerably higher than the DC *H*_on_ ~ 12.5 kOe located on the magnetic hysteresis curve at *T* = 20 K, overcoming the upper edge of the RST (see Fig. 2). Thus, in the used AC conditions, the vortex system is ordered in the RST domain. The striking result is the absence of any significant feature on the *m*′(*T*) variation which could be related to the DC *J*_c_(*T*) peak temperature *T*_M_ ~ 20 K from Fig. 5a, or, through (1), to the structural transition temperature *T*_RST_(*H* = 25 kOe) ~ 18 K (see Fig. 4). This means that the DC *J*_c_(*T*) peak at *T*_M_ ~ 20 K from Fig. 5a does not correspond to the RST in the elastic pinning regime. The effect of the elastic compliance of the BVG to the pinning landscape at the RST appears to be too small to generate the SMP. For random pinning, an efficient accommodation of vortices to the pinning centres necessitates the presence of dislocations in the vortex system. The *m*(*H*) shoulder can easily be generated by a precipitous, pinning-induced vortex-system disordering triggered by the *C*_sq_ softening at *H*_RST_, where one has a transition between the rhombic BVG and a partially dislocated (disordered) square BVG. The proliferation of dislocations at *H*_RST_ is favoured, since *H*_RST_ is close to *H*_on_, where the energy balance relation should be fulfilled.

**Figure 6:**
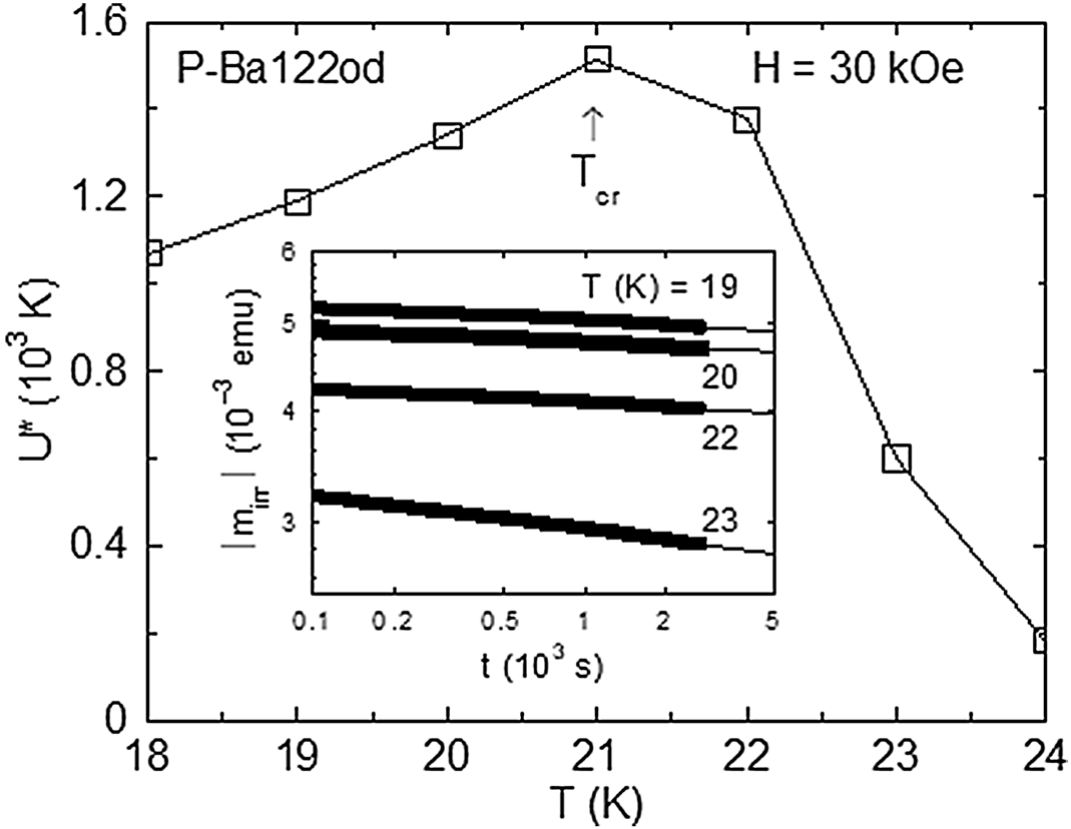
Inset: Time *t* dependence of the absolute value of the irreversible DC magnetic moment ∣*m*_irr_∣ in log–log scales for P-Ba122od in *H* = 30 kOe (applied in ZFC conditions) at several temperatures *T* ≥ 18 K. In this representation, the relaxation curves are linear, with the slope of the linear fit (the continuous line) supplying the normalized magnetization relaxation rate *S* =  − dln(∣*m*_irr_∣)/dln(*t*) and the corresponding normalized vortex-creep activation energy U* = *T*/*S*. Main panel: The resulting U*(*T*) variation exhibits a maximum at the creep-crossover temperature *T*_cr_, signalling the vortex system disordering across the second magnetization peak.

The pinning-induced vortex system disordering continues above the RST range, as indicated by the analysis of DC magnetization relaxation. The inset of Fig. 6 illustrates the relaxation time *t* dependence of the absolute value of the DC irreversible magnetic moment ∣*m*_irr_∣ in log–log scales for P-Ba122od at several temperatures in *H* = 30 kOe (applied in ZFC conditions). The considered temperature interval is above the RST domain, where the history effects are negligible. As known, when the relaxation time window *t*_w_ is moderate, in the representation from the inset of Fig. 6 the relaxation *m*_irr_(*t*) curves are linear, with the slope of the linear fit supplying the normalized magnetization relaxation rate *S* =  − dln(∣*m*_irr_∣)/dln(*t*) and the corresponding normalized vortex-creep activation energy U* = *T*/*S*^43,44^. For a given *H* well below the irreversibility line (where the pinning potential is not reduced significantly by thermal vortex fluctuations) and a fixed *t*_w_, the U*(*T*) variation obtained in the framework of the general vortex creep equation is approximated by

3 $$ U*\left( T \right)\sim U_{{\text{c}}} + pT{\ln}\left( {t_{{\text{w}}} /t_{0} } \right), $$

where *U*_c_ represents the characteristic pinning energy, *p* is the vortex creep exponent (positive in the case of an elastic vortex creep process^45^ and negative (around − 1/2^9^) for plastic creep, whereas *t*_0_ is the macroscopic time scale for creep^23^. The resulting U*(*T*) variation, plotted in the main panel of Fig. 6, exhibits a maximum at the creep-crossover temperature *T*_cr_, where, according to (3), the creep exponent changes sign, signalling the vortex system disordering across the SMP. This sign changing is possible through an increase of the characteristic pinning energy *U*_c_, owing to a better accommodation of vortices to the pinning centres in a disordered vortex phase. The applied field *H* = 30 kOe is close to the midpoint between *H*_on_ and *H*_p_ at *T* = *T*_cr_ (see Fig. 3), which is expected using global magnetic measurements^46^.

The existence of a maximum in U* (Fig. 6, main panel) means a minimum of the normalized magnetization relaxation *S* = *T*/U*, and it is tempting to locate the RST at this *S* minimum^32^. However, as it can be seen in Fig. 3, there is no maximum in ∣*m*∣ at *H* = 30 kOe on the magnetic hysteresis curve registered at *T* = *T*_cr_ = 21 K. Similarly, since the long-time relaxation measurements^25^ and structural investigations using muon-spin rotation and small-angle neutron scattering experiments^47,48^ showed that at the peak field *H*_p_ the vortex system is completely disordered, the identification of the RST line with *H*_p_(*T*) is ruled out.

Finally, the absence of the *m*(*H*) shoulder in the case of optimally doped and underdoped single crystals (the inset of Fig. 1 and^31^) can easily be explained in terms of the pinning-induced disordering of the vortex system across the SMP. A stronger pinning will shift *H*_on_ well below the intrinsic *H*_RST_(*T*) line, and the RST cannot manifest in a significantly disordered vortex system.

## Conclusions

In summary, we investigated the magnetic response of overdoped BaFe_2_(As_1−*x*_P_*x*_)_2_ single crystals, to clarify the relationship between the second magnetization peak and the characteristic structural rhomb-to-square transition of the Bragg vortex glass in the fourfold symmetric superconductors. It was found that the (elastic) RST does not generate the SMP, but the RST can influence the pinning-dependent SMP onset field when this is close to the intrinsic RST line, through the appearance of a shoulder on the magnetic hysteresis *m*(*H*) curves. The temperature evolution of the *m*(*H*) shoulder leads to the intersection of isothermal *m*(*H*) curves (a rare phenomenon), and, consequently, to a peak in the temperature variation of the DC critical current density *J*_c_(*T*). However, this particular peak does not appear in the temperature variation of the screening current in AC magnetic measurements where the vortex system is dynamically ordered in the RST domain. This emphasizes the essential role of vortex dislocations for an effective accommodation of the vortex system to the pinning landscape and the occurrence of the SMP.

We conclude that the observed DC *m*(*H*) shoulder is the effect of a precipitous pinning-induced proliferation of dislocations when the BVG elastic squash modulus softens at the structural transition between a rhombic and a partially disordered square BVG. The pinning-induced vortex-system disordering continues above the RST domain (as indicated by the magnetization relaxation results), and represents the basic SMP mechanism for the fourfold symmetric superconductors, as well. The absence of a notable effect of the RST on the SMP in the case of optimally doped and underdoped P-Ba122 single crystals can easily be explained in terms of the pinning-induced disordering of the vortex system across the SMP. The stronger pinning exhibited by optimally doped and underdoped P-Ba122 single crystals shifts the onset field well below the structural transition field, and the RST cannot manifest in a significantly disordered vortex system.

## Methods

**Figure 7:**
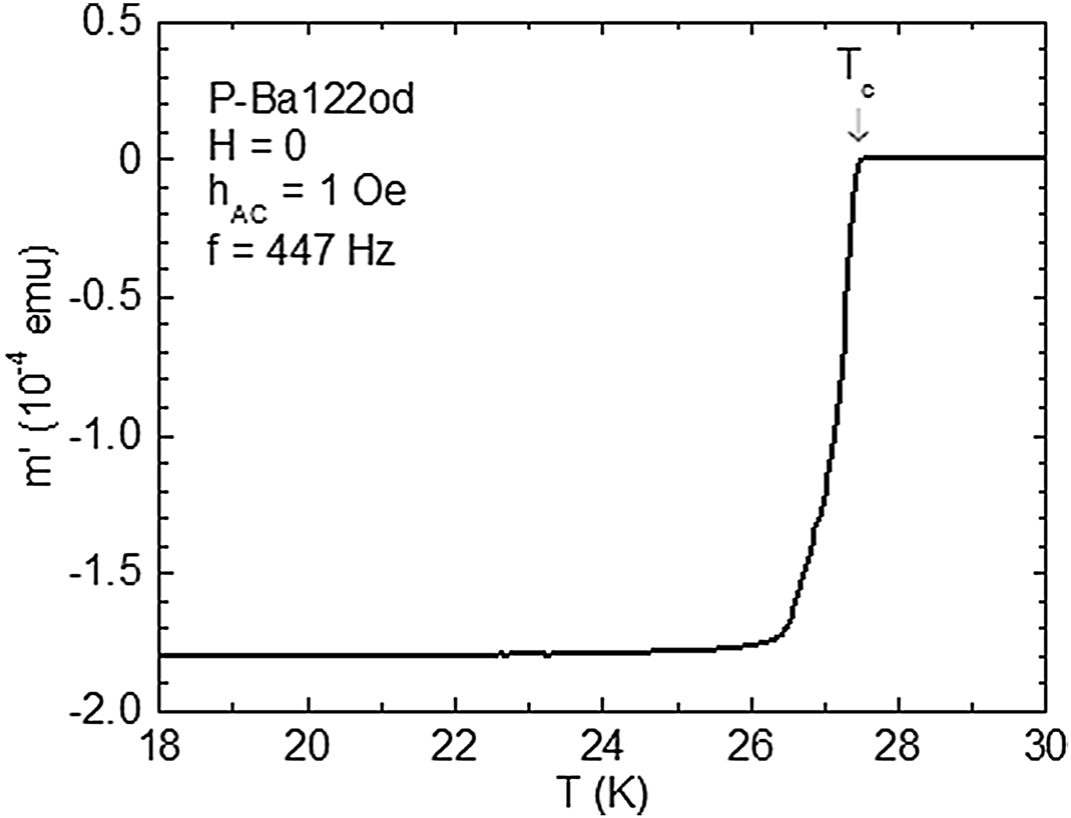
Temperature variation of the in-phase (screening) component *m*′ of the AC magnetic moment for P-Ba122od in *H* = 0 registered in increasing temperature with *h*_AC_ = 1 Oe and *f* = 447 Hz. The critical temperature *T*_c_ has be taken at the onset of the diamagnetic signal, and the width of the diamagnetic transition is around 1 K, reflecting the good quality of the thoroughly investigated single crystal.

The improved quality of iron-based superconducting single crystals^49,50^ made possible the observation of ordered vortex phases^34,50,51^. It is now established that the overdoped 122-type specimens exhibit a relatively weak, point-like *δT*_c_ pinning^52^ and a pronounced SMP develops^31^. The P-Ba122 single crystals analysed in this work have been grown by the Ba_2_As_3_/Ba_2_P_3_-flux method^53^ at the Institute of Physics, Chinese Academy of Sciences. They have been selected by the width Δ*T* of the diamagnetic transition in the *m*′(*T*) variation registered with *H* = 0, *f* = 447 Hz, and *h*_AC_ = 1 Oe, as illustrated in Fig. 7 for P-Ba122od, where Δ*T* ~ 1 K. The measured samples are square-shaped (with the side *l* ~ 1.7 mm), with the thickness *t* (in the direction of the crystallographic *c* axis) of ~ 50 μm.

The DC and AC magnetic fields have been oriented along the *c* axis, when the demagnetization factor^54^ *D* ~ 0.9. Thus, the AC field amplitude *h*_AC_ at the sample edge is enhanced by a factor 1/(1 − *D*) ~ 10. The DC critical current density *J*_c_ (in A/cm^2^) has been determined as 60 |*m*_irr_|/*l*^3^*t*^36^, with *l* and *t* in cm, and the irreversible magnetic moment *m*_irr_ = (*m*_+_ − *m*_−_)/2, with *m*_+_ (*m*_−_) representing the magnetic moment (in emu) measured in increasing (decreasing) *H*. When the reversible magnetic moment is negligible, |*m*_irr_| can be approximated by *m*_−_.

The DC *m*(*H*) curves were usually obtained with a commercial Quantum Design Magnetic Property Measurement System (MPMS), whereas the AC magnetic moment was registered with a Physical Property Measurement System (PPMS). The presented AC results for finite *H* correspond to *h*_AC_ = 10 Oe, and the frequency *f* = 447 Hz. The relatively high *h*_AC_ and the strong demagnetization effects allow attaining the nonlinear AC regime with a large AC critical state penetration far below the DC irreversibility line of overdoped P-Ba122 single crystals.

The temperature and DC magnetic field setting followed the zero-field cooling protocol (ZFC), obtained by fast cooling the sample (~ 10 K/min) in *H* = 0 from above *T*_c_ down to *T* < *T*_c_, then applying *H*, and measuring *m*(*H*) or the AC magnetic signal in increasing temperature at a slow rate (~ 0.05 K/min). For comparison, the field cooling on cooling procedure (FCC) has also been used, with *H* applied above *T*_c_, and then measuring the AC magnetic moment in decreasing *T* at the slow rate. Well below the irreversibility line, the contribution of the reversible magnetic moment to the DC *m*(*H*) curves is negligible. However, in the case of magnetization relaxation data registered over a large temperature interval, the irreversible magnetic moment *m*_irr_ has been taken into account.

## Data Availability

The data sets that support the findings in this study are available from the corresponding author upon reasonable request.
